# Biosynthesis of Metal Nanoparticles: Novel Efficient Heterogeneous Nanocatalysts

**DOI:** 10.3390/nano6050084

**Published:** 2016-05-05

**Authors:** Jose M. Palomo, Marco Filice

**Affiliations:** 1 Departament of Biocatalysis, Institute of Catalysis (CSIC), Marie Curie 2, Cantoblanco, Campus UAM, 28049 Madrid, Spain; 2 Advanced Imaging Unit, Spanish National Research Center for Cardiovascular Disease (CNIC), 28049 Madrid, Spain; mfilice@icp.csic.es

**Keywords:** metal nanoparticle, biosynthesis, peptides, sugars, proteins, heterogeneous catalysis

## Abstract

This review compiles the most recent advances described in literature on the preparation of noble metal nanoparticles induced by biological entities. The use of different free or substituted carbohydrates, peptides, proteins, microorganisms or plants have been successfully applied as a new green concept in the development of innovative strategies to prepare these nanoparticles as different nanostructures with different forms and sizes. As a second part of this review, the application of their synthetic ability as new heterogonous catalysts has been described in C–C bond-forming reactions (as Suzuki, Heck, cycloaddition or multicomponent), oxidations and dynamic kinetic resolutions.

## 1. Introduction

Nanotechnology has experimented a tremendous rise in the last decade [1,2,3,4]. In particular, the design of nanoparticles (NPs) has represented a very promising strategy alternative to conventional processes, especially of great application in environmental and biomedical problems (such as drug delivery, imaging, *etc.*) [5,6,7,8].

However, the field of nanocatalysis focused on the use of nanoparticles ashomogeneous or heterogeneous catalyst has been growth during the last years, [9,10,11,12,13]. The large surface-to-volume ratio of nanoparticles compared to bulk materials makes them attractive candidates for its use as catalysts. Especially the preparation and characterization of NPs from noble metals, which constitutes an important branch of heterogeneous catalysis in the chemical industry, represents an important challenge. The advantages of a very high superficial area make them excellent catalysts, reducing the amount of catalyst per gram of product making the process more sustainable. These NPs are synthesized by chemical methods in such a way to obtain good amounts, controlling the size and the form of the NPs [14,15,16]. However, in most cases hazardous conditions are used, toxic solvent, high amounts of energy (200 °C), which reduce a possible industrial implementation of this nanocatalyst. Most of these methods are still in the development stage and problems are often experienced with stability of the prepared nanoparticles, control of the crystal growth and aggregation of the particles [14,15,16]. Therefore the design of new synthetic approaches which considering an easy, rapid and sustainable strategy represents an important issue.

In recent years, a number of new green strategies have been described in literature. They are based on the capacity of biomolecules to induce the formation of these nanoparticles, sometimes even controlling the size and the structural form, and avoiding aggregation problems [17,18,19,20,21,22,23,24,25,26,27].

In this way, small molecules such as monosaccharides (glucose or galactose), or aminoacids and short peptides has been used as reducing agent for *in situ* creation of these metallic nanoparticles [17,18,19,20,21]. In addition, in a more precise way, the application of more complex biomolecules such as proteins or even microorganisms have been successfully applied to create nanoparticles and also hybrid systems with very high potential catalytic properties [23,24,25,26]. Bionanostructures, in which an enzyme is specifically encapsulated in a nanocluster or immobilized on biofunctionalized nanoparticles [10] are another category of catalysts with excellent features in cascade reactions. In particular, heterogeneous enzyme−metal nanoparticle nanohybrids with multiple catalytic activies are interesting in organic synthesis.

In this review, an overview of the recent advances on these new biosynthetic strategies and the use of the formed nanoparticles as catalyst in chemical processes is shown.

## 2. Synthesis of Metal Nanoparticles Induced by Glucosides

The most recent strategies targeting the synthesis of metallic nanoparticles have expected the introduction of green methodologies. In this sense, the use of glycosides avoids the necessity to apply toxic materials.

Thus, green synthesis of NPs induced by glucose and different glycopyranosides has been successfully reported [18,19,28]. In this case, very interesting results were found in the synthesis of gold nanoparticles (AuNPs) using eight different glucose derivatives [27].

**Figure 1:**
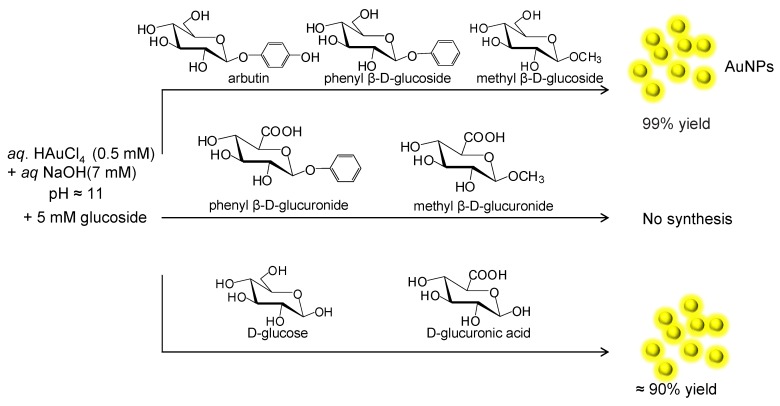
Synthesis of gold nanoparticles (AuNPs) induced by different glucose derivatives.

A room temperature and easy synthetic method of AuNPs was developed using auric acid, sodium hydroxide and different glycosides as reducing agent (Figure 1).

Eight sugar-containing reductants were used for comparison. *C-6* position of glycosides was oxidized to a carboxylic acid during the reduction of auric acid in the formation of AuNPs in the case of sugars substituted at the anomeric carbon/position (Figure 1). In the case of glucose or glucuronic acid (COOH in *C-6*), the NPs formation may be due to the oxidation of an aldehyde generated *via* anomerization. In this way, this explains why the synthesis in the presence of the glucuronic acid derivatives substituted at the anomeric position did not work (Figure 1).

**Figure 2:**
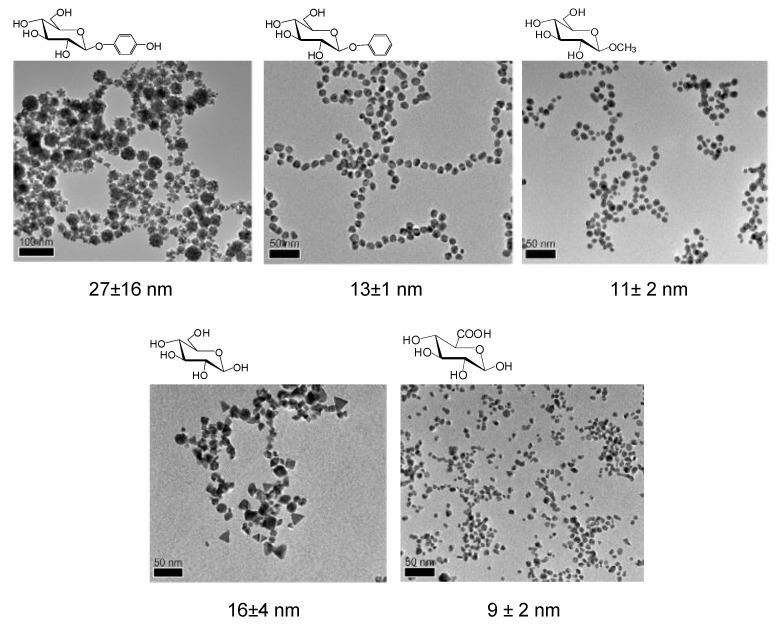
Characterization of the glucoside-induced AuNPs by high resolution transmission electron microscope (HRTEM).

Furthermore, significant differences in the final yield in AuNPs and especially the form and the size of the nanoparticles obtained by high resolution transmission electron microscope (HRTEM) (from 8 to 27 nm) depended on the sugar derivative (Figure 2).

The use of 1-phenyl or 1-methyl-β-glucopyranoside gave the highest synthetic yield (>99%) of homogeneous mono-dispersed round gold nanoparticles (13.15 nm, or 10.95 nm respectively), whereas using glucose or glucuronic acid the synthesized AuNPs showed multiple forms (16 and 8.8 nm). In the presence of arbutin, nanoparticles showed an amorphous form (27.4 nm) (Figure 2).

Therefore, this work shows how the size and the form of gold nanoparticles can be controlled by using different sugars as additives. This aspect is of special interest for example in the case of ultrasmall nanoparticles, where their colloidal stability can be affected by these small size differences, underscoring the importance of particle uniformity in nanomedicine [28].

These strategies could be also extended with other metals and also using other sugars derivatives with different degree of hydrophobicity by substitution in the anomeric and other positions.

In a recent approach, AgNPs were synthesized using aqueous extract of *Clerodendron serratum* leaves, which have high contents of polyphenol glycosides and quercetin 3-*O*-β-d-glucoside [29].

**Figure 3:**
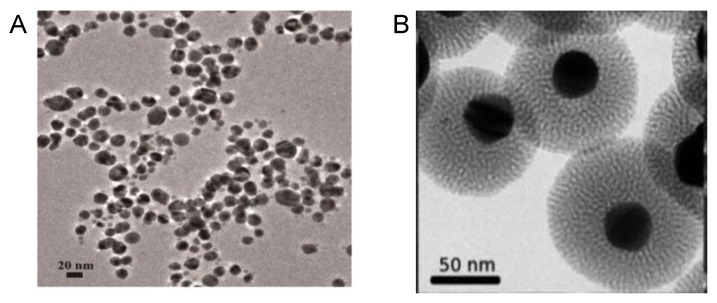
TEM image of different AgNPs. (**A**) AgNPs; (**B**) Mesoporous silica-coated silver nanoparticles (Ag@MSN) coated on a silicon substrate.

This glucose derivative caused the reduction of the silver ions of silver nitrate in 30 min at room temperature, forming spherical AgNPs with the size range of 5–30 nm (Figure 3A). In addition, the strategy of using glycosides has been successfully used in the preparation of mesoporous nanostructures [30].

Mesoporous silica-coated silver nanoparticles (Ag@MSN) have been prepared by a two mild step synthesis. Glucose was used as reducing agent for silver ions whereas arginine was used for the formation of silica. The nanostructure presented single Ag nanoparticles as cores of diameter *ca.* 30 nm surrounded a silica shell (thickness of *ca.* 30–40 nm), with a total average particle size (*ca.* 110 nm) (Figure 3B).

Another interesting example is the creation *in situ* of glycosylated functionalized gold nanoparticles [31].

**Figure 4:**
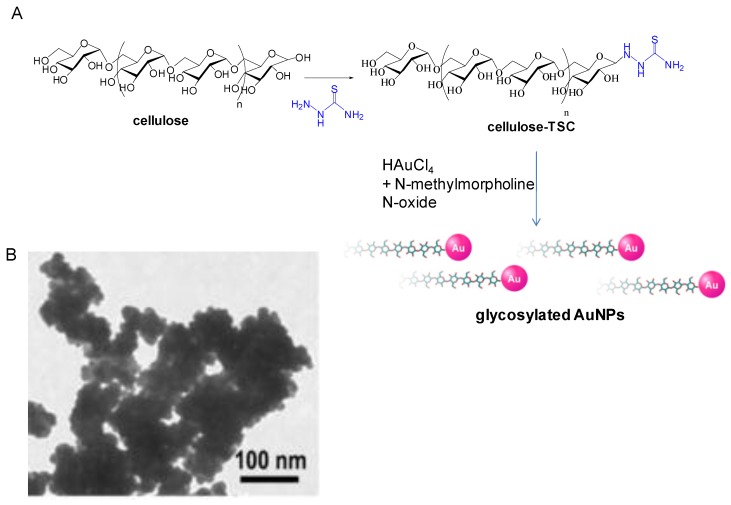
Synthesis of glycosylated AuNPs. (**A**) Synthetic scheme; (**B**) TEM image of glyco-AgNPs.

In this case, the glycoside acted as reducing agent but also is a specific moiety for particle functionalization. A glycopolymer (cellulose) was activated in the anomeric position by thiosemicarbazide producing glucoside thiosemicarbazone (Figure 4A). This activated sugar combined with an aqueous *N*-methylmorpholine *N*-oxide (NMMO)-a molecule which permits the solubilization of the water-insoluble cellulose- and a dilute aqueous HAuCl solution finally producing glyco-AuNPs. TEM analysis confirmed the formation of AuNP aggregates with primary sizes of *ca.* 10–20 nm in diameter (Figure 4B). Same protocol was used to successfully synthesize glyco-AgNPs with distribution size of *ca.* 5–30 nm diameters.

The combination of NMMO-mediated GNP synthesis and immobilization of sugar reducing ends to an Au° matrix, allowed the design of a diverse array of carbohydrate-GNP conjugates by tailoring the functional sugars, e.g., cellulose, chitin, chitosan, maltose and lactose [18].

## 3. Biosynthesis of Metal Nanoparticles by Peptides

The synthesis of biocompatible metal nanoparticles can be performed by using peptides as multifunctional reagents (reducing and capping agents) under mild conditions [32,33,34,35,36]. The large diversity of peptides available provides a new opportunity to organize, interact, and direct the shape, size, and structure evolution of the metal nanoparticles in more varied and innovative ways. In some cases these peptides also show the capability to reduce metal ions and to template the crystal growth of the metal nanoparticles [37].

**Figure 5:**
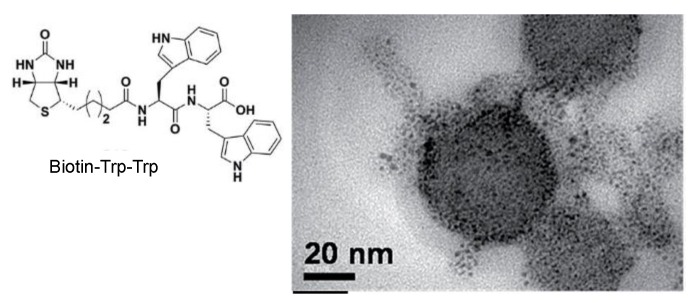
Self-organized AuNPs on the surfaces of biotin-Trp-Trp scaffold.

The use of a short conjugated peptide, such as a biotinylated di-tryptophan peptide was applied by Mishra and coworkers for the one-pot synthesis of stable gold nanoparticles [32]. The tryptophan dipeptide stabilized the NPs generated with an average size between 4 and 6 nm. Furthermore, self-assembled superior and ordered nanostructures of variable size were afforded where the AuNPs were scattered inside the biotinylated spherical scaffold in a controlled manner (Figure 5).

**Figure 6:**
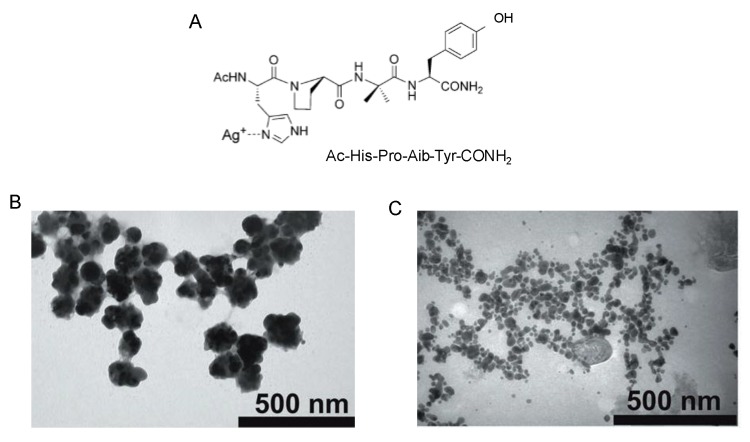
Synthesis of AgNPs. (**A**) Peptide structure; (**B**,**C**) TEM pictures Ag^+^-peptide complex after different irradiation times, *t* = 30 s and *t* = 30 min, respectively.

In another strategy, Giese *et al.* have recently described the synthesis of AgNPs under electron transfer conditions [33]. Ag^+^ ions are bound by a peptide including a histidine (Figure 6A) as the Ag-binding amino acid, and a tyrosine as a photo inducible electron donor. The presence of chloride ions was necessary for the final formation of AgNPs, which occur on AgCl microcrystals in the peptide matrix. In this way, by controlling the irradiation times, the formation of Ag@AgCl/peptide nanocomposites with a sized of 100 nm at the beginning of the process was obtained, which are cleaved after time finally generating the AgNPs with a diameter of 15 nm (Figure 6).

**Figure 7:**
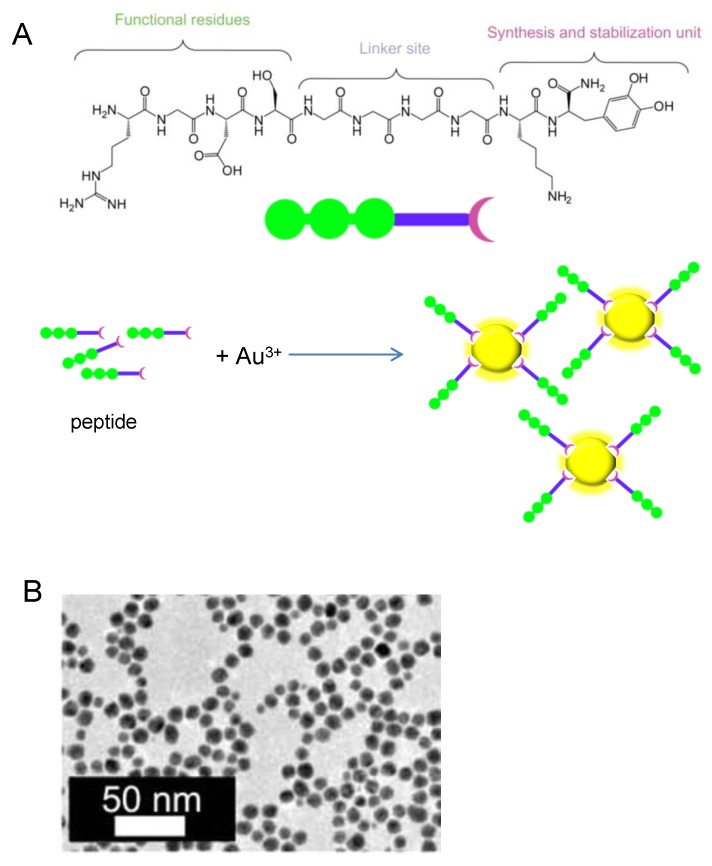
Synthesis of Biofunctionalized AuNPs. (**A**) Peptide and scheme of NPs formation; (**B**) TEM images of the AuNPs.

Tekinay and coworkers described the design and application of a multidomain peptide for single-step, size-controlled synthesis of biofunctionalized AuNPs (Figure 7) [34]. Size-controlled synthesis of AuNPs with this peptide was possible due to the 3,4-dihydroxy-l-phenylalanine (l-DOPA) functional group, a residue known for its reductive role. The authors showed DOPA coupled its oxidation to the reduction of Au (III) ions, thereby leading to the formation of biofunctionalized AuNPs. Hence, the DOPA-mediated peptide design enables concerted one-pot reduction, stabilization and functionalization of resulting AuNPs whereby no additional reagent or reaction is needed.

## 4. Bio-Inspired Synthesis of Nanoparticles by Proteins

**Figure 8:**
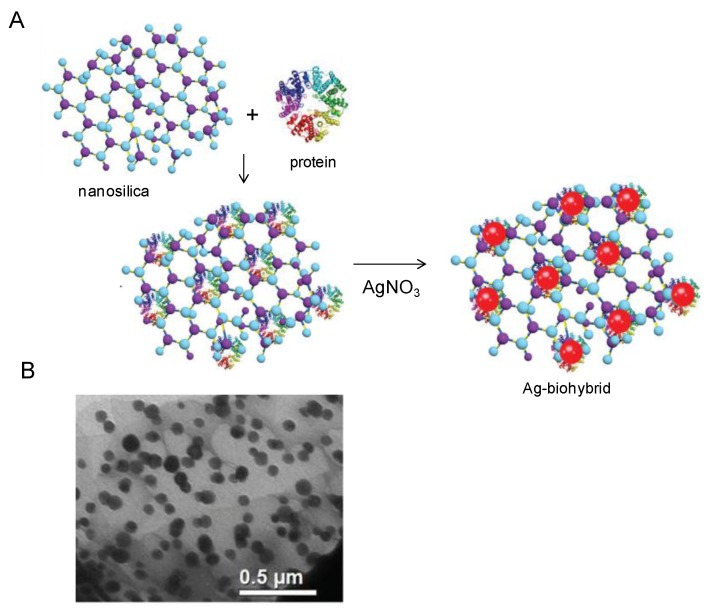
Synthesis of Ag-biohybrid. (**A**) Scheme of the formation of the nanostructure; (**B**) TEM of Ag-nanohybrid. Reproduced with permission from [39]. Copyright the Royal Society of Chemistry, 2013.

Among these strategies, the employment of microbial enzymes for nanoparticle synthesis is a new field with growing importance. In fact, considering that various enzymes have different capacities for synthesis of nanoparticles in a wide set of shapes and sizes, it is very important to find suitable enzymes for such purposes and improve the method for optimal nanoparticle production. Conversely, nanoparticles obtained by cell-based methods forcefully bind to the microbial biomass resulting in high-cost laborious steps of separation and purification of nanoparticles from microbial cells. In this line, many examples have been reported in literature. For example, Cholami-Shabami *et al.* developed a cell-free viable approach for synthesis of gold nanoparticles using NADPH-dependent sulfite reductase purified from *Escherichia coli*. [38] Highly stable gold nanoparticles were produced by reductive process after application of the sulfite reductase to an aqueous solution of AuCl_4_^−^. The enzymatically synthesized gold nanoparticles showed strong inhibitory effect towards the growth of various human pathogenic fungi [38]. Another interesting approach was developed by Kas and coworkers permitting the achievement of nanosilica-supported Ag nanoparticles by means of a biosynthetic protocol (Figure 8) [39]. After immobilizing a protein extract proceeding from *Rhizopus oryzae* on the surface of a nanosilica structured support, the authors carried out the synthesis of AgNPs using this biohybrid as host for the growth of AgNPs on its surface. In the proposed *in situ* synthetic process, the Ag^+^ ions-proceeding from an AgNO_3_ solution as metal precursor- were considered to be rapidly adsorbed on negatively charged protein surfaces through electrostatic interaction. The reduction of Ag^+^ ions and the subsequent formation of AgNPs was due to the electron transfer between the metal ions and the functional groups of proteins.

**Figure 9:**
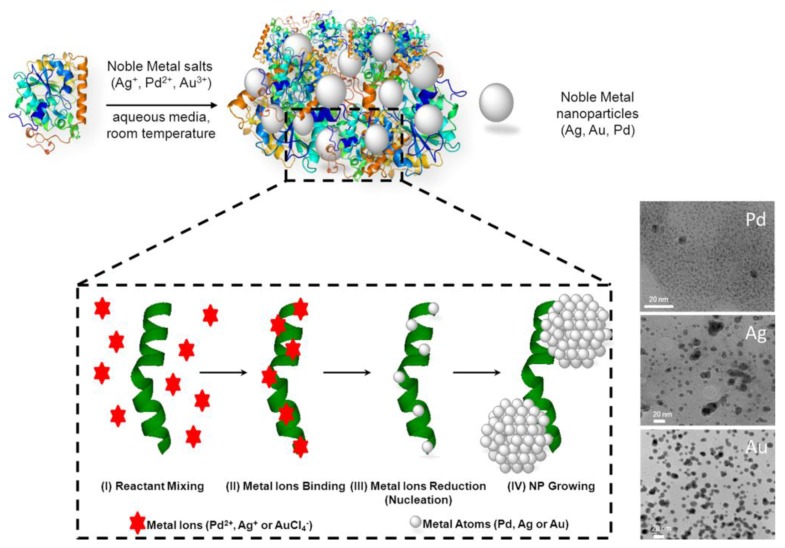
Preparation of metal bionanohybrids.

Following this research line, a novel type of heterogeneous hybrid nanocatalysts, composed by metal NPs embedded in an enzymatic net, was generated *in situ* under very mild reaction conditions from the simple mixture of lipase from *Candida antarctica* fraction B (CAL-B) with a homogeneous aqueous solution of a noble metal salt (Ag^+^, Pd^2+^, or Au^3+^) (Figure 9) [22,40].

This new hybrid nanocatalysts combines metallic and enzymatic catalytic activitie. The use of an enzyme in the methodology permitted the generation of small metal NPs (e.g., around 2 nm core size for Pd NPs) without the need for any external reducing agent, exploiting the reductive ability of the biomacromolecule (biomineralization), which moreover remains catalytically active at the end of the synthesis [22,40]. Based on the same general bio-based strategy, Das *et al.* reported an interesting biosynthetic route for cost-effective productions of various metal NPs (Pd, Pt, and Ag) on the surface of fungal mycelia [41]. The metal NPs were synthesized through an electrostatic interaction of metal ion precursors, followed by their reduction to nanoparticles by surface proteins finally decorating the mycelia surface in a homogeneous way. It results worth of note as, by means of this strategy, the size and shape varied depending on the metal NPs. In fact, “flower”-like branched nanoparticles were obtained in the case of Pd and Pt, while Ag produced spheroidal nanoparticles, this structural characteristic is a key-element of their catalytic activity which is assessed in hydrogenation and Suzuki C–C coupling reactions in aqueous solution [41].

Even engineered proteins were revealed to be useful in the synthesis of precise and highly functionalized metal nanoparticles. For example, a small variant of protein A has been used as biotemplate in the one-step synthesis and biofunctionalization of AuNPs [42]. This biotemplate is composed by a thiolate ligand capable of interacting with the AuNP surface and controlling the nanoparticle nucleation and growth, thus allowing the nanoparticle size to be finely tuned. This crucial feature clearly resulted as key-advantage of this approach, which allow for high-quality AuNPs to be obtained in the water phase, and therefore avoiding the transfer from organic solvents, which usually results in a lack of long-term stability [42].

Moving a step over the well-known strategy based on the creation of metallic nanoparticles inside the cavity of hollow protein (*i.e.*, ferritin), Jang and coworkers described the synthesis of thin-walled (*ca.* 40 nm) SnO_2_ nanotubes functionalized with catalytic Pt and Au nanoparticles via a protein templating route [43]. After the creation of metal NPs inside the cavity of an apoferritin template via NaBH_4_ reductive strategy starting from metal salt precursors, as the prepared hybrids catalysts were used to decorate both the interior and exterior surfaces of the thin-walled SnO_2_ nanotubes. After calcination, the protein cage was eliminated leaving a well-dispersed layer of catalytic metal nanoparticles immobilized on nanotubes surface. Such a uniform surface distribution, resulting from the repulsion between the proteic cages before their calcination, granted a final very high surface area-to-volume ratio leading to superior catalytic performances for example in gas sensing [43].

## 5. Biosynthesis of Metal Nanoparticles by Microorganisms

Apart of the use of small molecules or even proteins, the use of entire biological units as prokaryotic or eukaryotic microorganisms have been employed for the preparation of nanoparticles of different metals (Au, Ag, Cd, Pt, Zn, Fe_3_O_4_) under moderate pressures and temperatures [44,45,46,47].

Microorganisms are capable of adsorbing and accumulating metals. They also secrete large amounts of enzymes, which are involved in the enzymatic reduction of metals ions [48,49]. Microbial synthesis of metallic nanoparticles can take place either outside or inside the cell [44], producing metal NPs, which have characteristic features similar to nanomaterials, which are synthesized chemically [14]. The localization, size or shape of the nanoparticles depend on the microorganism specie used [44].

In this way, the production of metal nanoparticles by fungi is one of the most successful strategies [42,50,51,52,53].

For example, in one of the cases, the fungus *Aspergillus japonica* was used for the reduction of Au (III) into Au NPs. Spherical and well distributed on fungal mycelia particles were found. The size of the particles ranges predominantly between 15 and 20 nm. Furthermore, the nanoparticles were simultaneously immobilized on the fungus surface creating a heterogeneous hybrid with interesting catalytic properties [50].

Another example of the use of fungus was described by Loshchinina and coworkers [51]. The authors described the synthesis of AuNPs by the fungus *Basidiomycete lentinus edodes*. TEM experiment demonstrated the formation of spherical Au(0) nanoparticles inside the mycelia cells mostly of 5 to 15 nm with minor part of 30 to 50 nm diameter. An Au distribution map was obtained that supported these electron-dense formations to be intracellular Au nanoparticles.

This is also the first time that fungal intracellular phenol-oxidizing enzymes (laccases, tyrosinases, and Mn-peroxidases) have been involved in Au reduction to give electrostatically stabilized colloidal solutions.

Also the preparation of AuNPs has been described by Gupta and coworkers using in this case the fungus *Trichoderma* sp. [52]. The biosynthesis of the nanoparticles was rapid at 30 °C using cell-free extract of the *Trichoderma viride*, producing extracellular AuNPs with particle size of 20–30 nm. Using *Hypocrea lixii* the synthesis was similar but at 100 °C, obtaining smaller nanoparticles (<20 nm).

**Figure 10:**
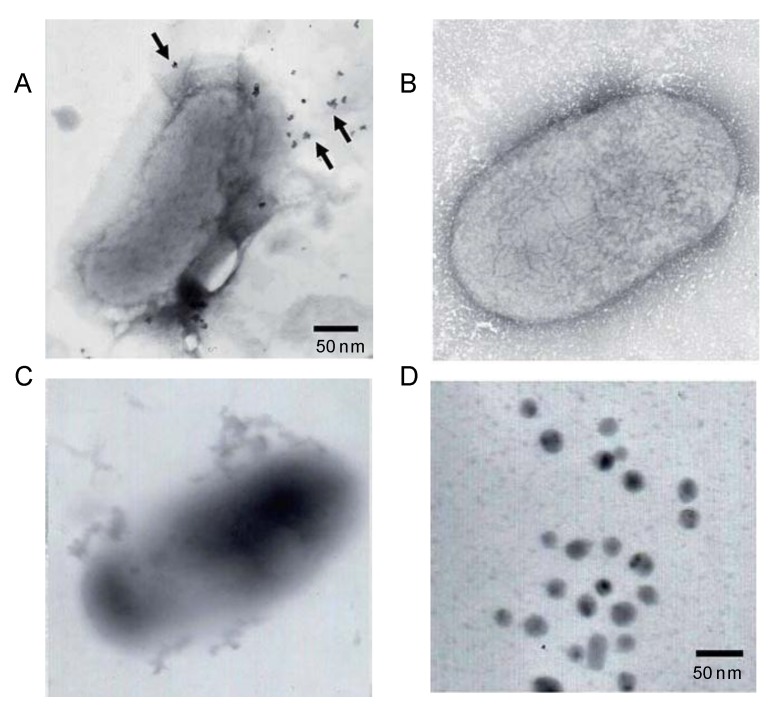
Eumelanine from tyrosinase induced the synthesis of gold nanoparticles. (**A**) TEM image of *R. etli* cell in the presence of 3-(3,4-dihydroxyphenyl)-l-alanine (l-DOPA) and Au ions; (**B**) TEM image of *R. etli* cell in the presence of Au ions; (**C**) TEM image of *R. etli* cell in the presence of l-DOPA; (**D**) TEM of the synthesized AuNPs. Reproduced with permission from [53]. Copyright the Royal Society of Chemistry, 2014

The use of recombinant *E. coli* expressing a tyrosinase from *Rhizobium etli* has been described as interesting green strategy to synthesize gold nanoparticles [54]. Tyrosinase is an important enzyme in biology involved in production of melanin. The catalytic function is the oxidation of l-tyrosine to 3-(3,4-dihydroxyphenyl)-l-alanine (l-DOPA) and further to dopaquinone and melanin. In particular, eumelanin–natural pigment, which contains carboxyl, amine, hydroxyl groups, quinone and semiquinone groups–was used as agent to reduce the metals ions. In the presence of l-DOPA and gold ions, exogenous AuNPs were formed (Figure 10A). The absence of l-DOPA failed in the formation of AuNPs, demonstrating that the presence of eumelanin (generate by the enzyme with l-DOPA) is critical for the nanoparticles formation (Figure 10B). In the absence of gold ions, the transformation of l-DOPA to eumelanin was observed (Figure 10C). The TEM analysis demonstrated that the AuNPs showed a particle size average of around 12 nm (Figure 10D). The strategy was successfully applied to other metals obtaining NPs with a particle size between 7 and 13 nm [53].

## 6. Biosynthesis of Metal Nanoparticles by Plant Extracts

Another strategy used for preparation of biosynthetic green metal nanoparticles is based on the use of extract of different plants. These contain a wide amount of natural products such as polyphenylols, alkaloids, flavonoids and terpenoids (reducing and stabilizing agents), which induced the final formation of the nanoparticles. An interesting review article about that has been published recently by Banerjee and coworkers [24] and no extended description of this strategy will be present here.

However, we would like to emphasize two very recent works in the preparation of mono and bimetallic nanoparticles [12,54].

**Figure 11:**
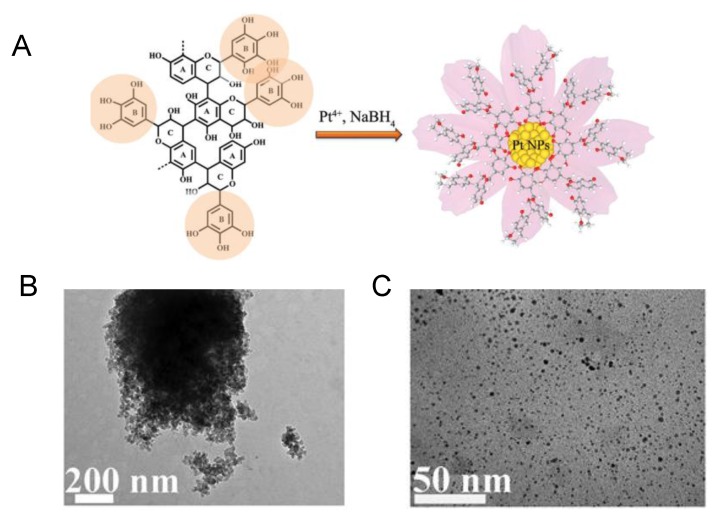
Synthesis of Pt nanoparticles induced by black wattle tannin (BWT). (**A**) Scheme of the formation of PtNPs by BWT; (**B**) TEM image of synthesized PtNPs using 2 mg of BWT; (**C**) TEM image of synthesized PtNPs using 15 mg of BWT. Reproduced with permission from [54]. Copyright the Royal Society of Chemistry, 2016.

The first describes the synthesis of platinum nanoparticle (PtNP) aqueous colloid by utilizing black wattle tannin (BWT), a typical plant polyphenol (Figure 11) [54]. The hydroxyl groups of this molecule act as reducing agent but also as stabilizers and protecting the PtNPs from deactivation caused by oxygen atmosphere. The aromatic framework of BWT prevents the aggregation of the nanoparticles in contrast with the use of the most hydrophilic organic small molecules. Stable heterogeneous metallic nanoparticles under mild conditions can be then obtained by this method. Different amount of BWT were tested showing that only when 15 mg of BWT was added, completely monodispersed Pt small nanoparticles (*ca.* 1.8 nm diameter size) were formed (Figure 11B).

**Figure 12:**
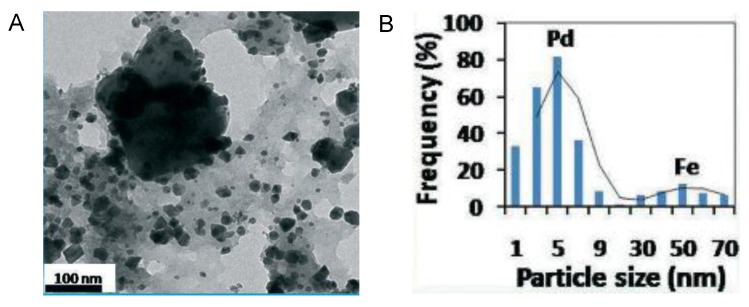
Characterization of Fe-Pd NPs synthesized by *Ulmus* *davidiana.* (**A**) TEM image of Fe-Pd NPs; (**B**) Particle size distribution histogram. Reproduced with permission from [12]. Copyright the Royal Society of Chemistry, 2015.

The second example refers to the synthesis of Fe, Pd and Fe-Pd bimetallic nanoparticles using the medicinally potent aqueous bark extract of *Ulmus* *davidiana* [12]. As shown in the previous case, the polyols present in the plant were responsible for reducing and capping of the nanoparticles. The NPs preparation was achieved using aqueous solution of *Ulmus* adding firstly Fe_2_O_3_, PdCl_2_ or the bimetallic iron oxide and then the palladium chloride at 60 °C. The three sort NPs showed different diameter size determined by TEM. Spherical FeNPs showed 50 nm or PdNPs with 5 nm-sized were obtaining using a unique metal in the synthesis. On the bimetallic, the PdNPs were adsorbed on the outer surface of the FeNPs (Figure 12A). The distribution size were 3–7 nm of Pd clustered around 30–70 nm of FeNPs (Figure 12B).

## 7. Application of the Biosynthesized Metallic NPs as Nanocatalysts

Beside the description of the metal NPs preparation, the different methods presented above have been tested on the reduction of *p*-nitrophenol to *p*-aminophenol as general model reaction in order to assess the catalytic properties of generated nanobiocatalysts.

As representative example, the catalytic constant (*k*) and the turnover frequency (TOF) of that reaction using the CAL-B-Pd biohybrid were calculated, retrieving excellent *k* and TOF values (0.6 min^−1^ and almost 150 min^−1^, respectively). The TOF value was the highest described in the literature for this reaction at that moment [22].

In parallel to the model aryl-amine synthesis, some of the previous synthesized heterogeneous catalysts have been successfully used in different typology of complex reactions.

**Figure 13:**
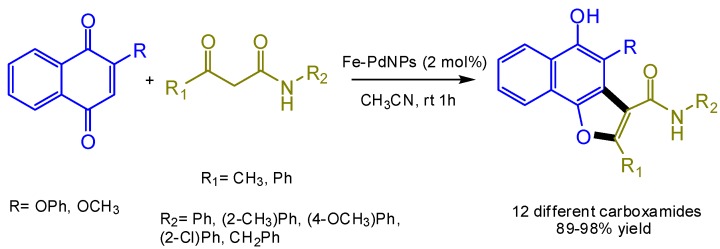
Synthesis of substituted carboxamides catalyzed by Fe-Pd NPs nanocatalyst.

Thus, the bimetallic nanoparticles formed by Fe and Pd represented a catalyst magnetically recyclable and reused in [3 + 2] cycloaddition reaction [12] (Figure 13). These Fe-Pd NPs displayed better catalytic activity, with final isolated yields from 89% to 98% for the synthesis of 12 different naphtha[1,2-b]furan-3-carboxamides and benzofuran-3-carboxamides compared to their respective monometallic nanoparticles (Figure 13).

Advantages of this heterogeneous nanocatalyst were an easy recovery using an external magnetic field, and recycling (maintaining almost complete activity after 5 times).

**Figure 14:**
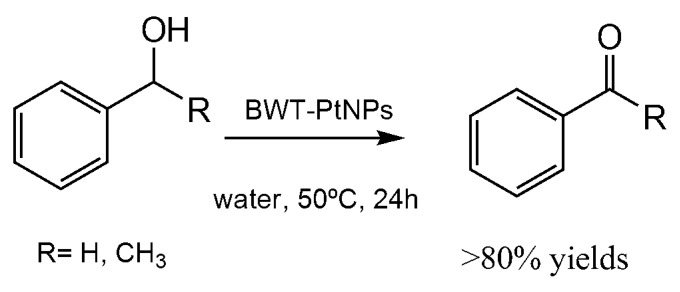
Oxidation of phenylalcohols by BWT-PtNPs.

Another interesting example of practical usefulness is represented by the BWT-Pt colloid catalyst [54]. This nanocatalyst showed interesting activity in a series of biphasic oxidation of aromatic alcohols and aliphatic alcohols under mild aerobic conditions in aqueous media. The results were from moderate yields, for alkyl compounds, to high yield, for aromatic alcohols. The best results were obtained in the oxidation of phenylmethanol and 1-phenylethanol with more than 80% yield of product (Figure 14). The nanocatalyst was very stable and no decrease in the oxidative activity was observed after seven cycles.

The synthesis of propargylamines by a multi-component reaction has been successfully catalyzed by means of the gold-NPs-fungal hybrid bionanocatalyst [46]. These bioNPs produced different propargylamines by an A3 coupling process, a very interesting synthetic way to produce heterocyclic frameworks [55].

**Figure 15:**
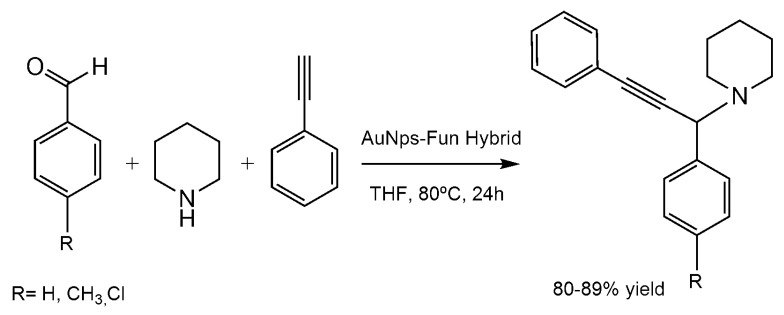
Multicomponent reaction catalyzed by AuNPs Hybrid.

The high versatility of the nanocatalyst was demonstrated and over 80% yields of various propargylamine derivatives were obtained using a variety of aromatic aldehydes (R = H, CH_3_, Cl) coupled with secondary amine and alkynes after 24 h (Figure 15). In addition, this heterogeneous hybrid showed good recyclability.

**Figure 16:**
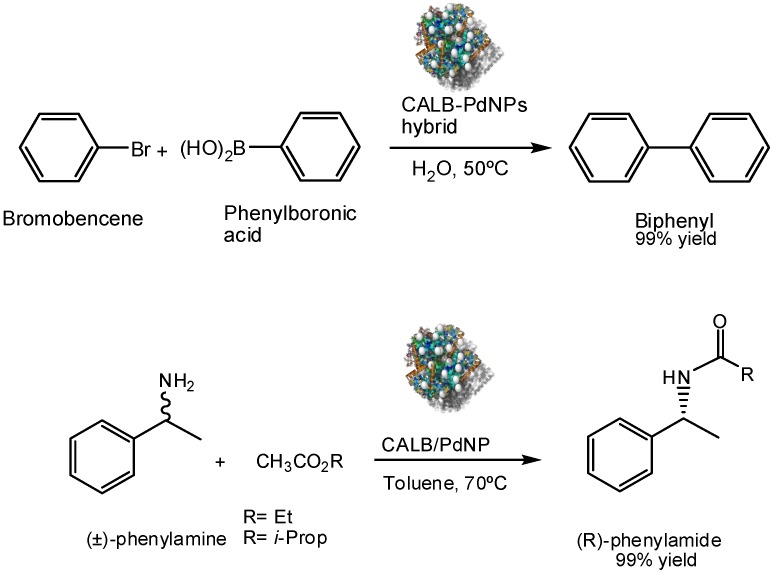
Catalytic applications of CAL-B-PdNPs hybrid.

The Pd-lipase bionanohybrid described before was successfully applied as excellent heterogeneous catalysts in C–C bond reaction [22]. This resulted in an extremely active Pd-catalyst in the Suzuki reaction, forming the biphenyl in >99% conversion using *ppb of catalyst* (Figure 16). Furthermore, the reaction was performed in pure water so making this organic reaction a green chemical process. A recyclable catalyst was obtained, used five times conserving almost the activity intact.

The particular advantage of this catalyst is that it conserved the native enzymatic activity, so it was successfully applied in the dynamic kinetic resolution of *rac*-phenylethylamine in organic solvent, a tandem catalytic process (both enzymatic and Pd catalysis acting at the same time) (Figure 16). The bionanohybrid quantitatively produced the enantiopure (*R*)-benzylamide with *ee* > 99%. Even in this case, the recyclability of the catalyst was excellent.

Finally, also the previously described “flower”-like branched Pd nanoparticles were used as heterogeneous catalyst in the Suzuki reaction of formation of biaryl with satisfactory results (99% conversion) [41].

## 8. Conclusions

In conclusion, this review showed the most recent different strategies used for the biosynthesis of metal nanoparticles. The application of biological entities represents an interesting and green solution for the environmentally friendly synthesis of these nanoparticles. The use of enzymes as biomolecule permits the design of more precise nanostructures for many interesting chemical applications and it allows combination of one or more metallic activities together with enzymatic catalytic ones (excellent for cascade processes). Therefore, the development of newer and more efficient bio-methodologies for the creation of nanobiohybrids will be a future issue. Together with that, the reviewed examples showed also the excellent catalytic application of these heterogeneous nanocatalysts demonstrating the tremendous potential of their use in organic synthesis.
